# Determinants of externally visible birth defects among perinatal deaths at Adama Comprehensive Specialized Hospital: a case-control study

**DOI:** 10.1186/s12887-024-04729-8

**Published:** 2024-04-20

**Authors:** Husen Aman, Seifadin Ahmad, Getahun Chala, Mekbeb Afework

**Affiliations:** 1 Department of Human Anatomy, College of Health and Medical Sciences, Haramaya University, Harar, Ethiopia; 2 Department of Public Health, Institute of Health, Ambo University, Ambo, Ethiopia; 3 Department of Medical Physiology, College of Health and Medical Sciences, Haramaya University, Harar, Ethiopia; 4 Department of Anatomy, College of Health Sciences, Addis Ababa University, Addis Ababa, Ethiopia

**Keywords:** Antenatal care, Birth defect, Congenital anomalies, Externally visible birth defect, Folic acid supplementation, Perinatal death

## Abstract

**Background:**

Birth defects (BDs) are the major causes of infant morbidity and mortality in both developed and developing countries. Regardless of their clinical importance, few studies on predisposing factors have been conducted in Ethiopia. However, due to a lack of advanced diagnostic materials, we only considered the externally visible BDs.

**Objective:**

To assess the determinants of externally visible birth defects among perinatal deaths at Adama Comprehensive Specialized Hospital.

**Methods:**

A retrospective unmatched case-control study design was conducted from November 01 to 30, 2021. The sample size was determined by Epi Info version 7 software considering sample size calculation for an unmatched case-control study. A total of 315 participants (63 cases, and 252 controls) were selected by simple random sampling. Data were collected by an open data kit (ODK) and transported to a statical package for social sciences (SPSS) version 26 software for analysis. The bivariate followed by multivariable logistic regression analyses were done to determine the factors associated with the BD.

**Results:**

This study showed that drinking alcohol during pregnancy (AOR = 6.575; 95% CI: 3.102,13.937), lack of antenatal care (ANC) follow-up during pregnancy (AOR = 2.794; 95% CI: 1.333, 5.859), having a history of stillbirth in a previous pregnancy (AOR = 3.967; 95% CI: 1.772, 8.881), exposure to pesticides during pregnancy (AOR = 4.840; 95% CI: 1.375, 17.034), having a history of BDs in a previous pregnancy (AOR = 4.853; 95% CI: 1.492, 15.788), and lack of folic acid supplementation during early pregnancy (AOR = 4.324; 95% CI: 2.062, 9.067) were significant determinants of externally visible BDs among perinatal deaths.

**Conclusion:**

In this study, alcohol use, exposure to pesticides, and lack of folic acid supplementation during pregnancy were identified as the major determinants of externally visible BDs among perinatal deaths. Thus, health education regarding the associated factors of BDs and their preventive strategies should be given to pregnant mothers.

## Introduction

Congenital anomaly (CA), birth defect (BD), and congenital malformation are synonymous terms defined as a structural or functional anomaly that occurs during intrauterine or after birth in life [1, 2]. It is thought to have occurred in both animals and humans as a result of a divine curse or evil in early human history. Some cultures still think that mothers who give birth to infants with BDs have communication with the devil or evil spirits [3]. However, the International Classification of Diseases (ICD) is stated CAs as congenital defects of body structure or function, likely to result in mental or physical handicap or death. In fact, a child with BDs can be born in any family including young, healthy, with no bad habits, and a normal pregnancy within any society [2].

A report from the World Health Organization (WHO) [4] stated that there are more than 6.3 million perinatal deaths occur per year worldwide. Of these, more than 2.64 million are stillbirths and 3 million are cases of early neonatal deaths. 98% of these deaths happen in developing countries. Birth defects are the 5th leading cause of years of potential life lost before age 65 and a major contributor to disabilities of a child [5]. The risks of BDs are greatest during the critical embryonic period (3rd to 8th week(s) of gestation), which is critical for fetal development. It is stated that approximately 303,000 newborns pass away yearly all over the world within thirty days because of BDs [1, 6, 7].

According to WHO estimation and the maternal and child epidemiology estimation group (MCEE), BDs were responsible for 11% of neonatal mortality in Ethiopia. Only 2.7% of women were prescribed folic acid during the protective period against neural tube defect (NTD), which was very low compared with 21% usage in Croatia, which had similar levels of planned pregnancy [8]. This contributes to an increase in the prevalence of BDs and their impact in developing countries such as Ethiopia. Birth defects can result in long-term physical, mental, visual, and auditory disabilities if not managed properly, with significant negative consequences for individuals and families, the health care system, and societies [1, 9].

Some BDs can be prevented by removing their risk factors and strengthening protective mechanisms. Thus, adequate dietary intake of vitamins (folic acid) and minerals, and avoiding harmful substances including tobacco, alcohol, heavy metals, pesticides, certain medications, and radiation are necessary for mothers to minimize the occurrence of the BDs [10, 11].

Although the prevalence of BDs is high in Ethiopia, there are few studies conducted on the determinants of externally visible BDs [10]. As a result, limited data are available at a local level on BDs for early intervention. This study could help in filling the gap of information on determinants of externally visible BDs among perinatal deaths at Adama Comprehensive Specialized Hospital (ACSH). In addition, it provides baseline data for future studies and public health measures. In this study, we only considered the externally visible BDs due to a lack of advanced diagnostic materials.

## Materials and methods

### Study setting and period

The study was conducted at ACSH from November 01 to 30, 2021. This hospital was selected based on patient load.

### Study design

A retrospective unmatched case-control study design was used to assess the determinants of externally visible birth defects among perinatal death.

### Source and study population

All perinatal deaths that occurred at ACSH were the source population of the study. All perinatal deaths that occurred between January 01 to December 31, 2020 at ACSH were the study population.

### Inclusion and exclusion criteria

The Stillbirths or neonatal deaths before the first 7 days of life with and without externally visible BDs were included as cases and controls, respectively. The Stillbirths or neonatal deaths after 7 days with and without externally visible BDs were excluded from the cases and controls, respectively.

### Sample size determination and sampling technique

The sample size was calculated using Epi Info version 7 software for unmatched case-control using the Fleiss formula with continuity correction factor and case-control study ratio of 1:4; confidence level of 95%, power of 80%, a significance level of 5%, and a proportion of controls exposed (Drank alcohol) 8.7% [12], assuming the minimum odds ratio to be detected was 3.125. Adding 10% incomplete medical cards, the calculated sample size was 315 (63 cases and 252 controls). The study samples were selected by a simple random sampling technique based on their hospital registration number.

### Study variables

**Dependent variable:** Externally visible birth defect.

**Independent variables:** Maternal age, Sex, Residency, Occupation, Deliveries, Number of previous pregnancies, History of abortion, Family history of congenital heart defects, History of maternal chronic illness, History of maternal alcohol intake, Previous history of maternal drug intake, Maternal health, and Diet and substance use during pregnancy.

### Data collection tools and technique

The data relating to the information of the study participants were collected from the medical cards and filled into checklist forms. The checklist form was the study instrument that had three sections: sociodemographic, behavioral, and reproductive and obstetric characteristics of perinatal death mothers. Two diploma and one BSc midwife was recruited from ACSH Medical College as data collectors. The data collectors and supervisor took the COVID-19 transmission prevention measures during the data collection period.

### Data quality control and management

The collected data were reviewed and checked for omissions, completeness, and consistency by the data collectors and principal investigator on daily bases during data collection time.

### Data analysis and interpretation

Data were collected by open data kit (ODK) and transferred to the statical package for social sciences (SPSS) version 26 software for analyses. Binary logistic regression analysis was done to observe the association of each independent variable to the dependent variable and variables with *p*-values of less than 0.25 were identified. The identified variables were entered into a multivariable logistic regression model to identify the independent factors associated with a BD. The 95% confidence interval (CI) and an associated factor with a *p*-value of less than 0.05 were considered statistically significant.

## Results

### Socio-demographic characteristics of the mother

Around 88.9% of mothers of cases and 85.3% of controls were between the ages of 15 and 35 years old. Almost half of the mothers of cases and controls were employed and unemployed, respectively. Over half of the mothers of cases (66.7%) and controls (53.6%) were urban residents. Statistically, there is a significant difference between case and control with regard to the religion of the mothers (*p* = 0.008) (Table 1).

**Table 1:** Socio-demographic characteristics of perinatal deaths with externally visible birth defects and their mothers as compared to the controls at ACSH, Adama, Ethiopia, 2020

Variable		Case (*n* = 63)	Control (*n* = 252)		*p*-value
		N (%)	N (%)	Chi-square	
Age of mothers	15–24	34(54%)	119(47.2%)	1.068	0.586
	25–35	22(35%)	96(38.1%)		
	> 35	7(11%)	37(14.7%)		
Maternal occupation	Student	9(14.3%)	26(10.3%)	2.98	0.225
	Unemployed	24(38.1%)	126(50%)		
	Employed	30(47.6%)	100(39.7%)		
Residence of mothers	Urban	42(66.7%)	135(53.6%)	3.511	0.061
	Rural	21(33.3%)	117(46.4%)		
Status	Alive Stillborn	20(31.8%) 43(68.2%)	87(34.5%) 165(65.5%)	0.173	0.677
Child’s gender	Male Female	42(66.7%) 21(33.3%)	122(48.4%) 130(51.6%)	6.73	0.0095

*Abbreviation* N, Frequency; n, Sample Size

### Reproductive and obstetric characteristics

Mothers who had a previous history of abortion were 28.6% in cases and 23% in controls, and 38.1% of cases and 13.1% of controls had a previous history of stillbirth. The majority of perinatal cases (58.8%) and controls (80.3%) were given birth at term or above, and 41.3% of cases and 29.8% of controls were given birth before 36 weeks. According to the findings of this study, the majority of women (84.1% of cases and 72.2% of controls) were given BD in the 1 to 4 birth order. Contraception was used by 68.3% of mothers who had a child with BDs, and 64.3% of mothers who had no child with BDs. Statistically, there is a significant difference between case and control with regard to the child’s gender (*p* = 0.0095) and the history of stillborn (*p* = 0.001) (Table 2).

**Table 2:** Reproductive and obstetric characteristics of perinatal death mothers at ACSH, Adama, Ethiopia, 2020

Variable		Case (*n* = 63)	Control (*n* = 252)	Chi-square	*p*-value
		N (%)	N (%)		
History of abortion	Yes	18(28.6%)	58(23%)	0.85	0.357
	No	45(71.4%)	194(77%)		
History of stillborn	Yes	24(38.1%)	33(13.1%)	21.25	0.0001
	No	39(61.9%)	219(86.9%)		
Gestational age	Term	27(42.9%)	140(55.6%)	3.57	0.170
	Preterm	26(41.3%)	76(30.2%)		
	Post-term	10(15.9%)	36(14.3%)		
Order of birth	1–4	53(84.1%)	182(72.2%)	3.77	0.52
	> 4	10(15.9%)	70(27.8%		
Contraception before pregnancy	Yes	43(68.3%)	162(64.3%)	0.349	0.555
	No	20(31.7%)	90(35.7%)		

*Abbreviation* N, Frequency; n, Sample Size

### Classification of the birth defects

All cases had NTDs. Of these, 24 had anencephaly with three umbilical hernias and one a cleft lip, 18 had hydrocephalus with two imperforate anus, and 5 had microcephaly with a chest deformity. There were also 6 Encephalocele, 5 spinal Bifida with one bilateral clubfoot, and 5 Meningomyelocele with one umbilical hernia.

### Determinants of externally visible birth defects

Maternal age, residence, childbirth status, mother’s religion, mother’s occupation, gestational age, contraception and tobacco use during pregnancy, and abortion were not statistically significant in binary logistic regression analysis. The variables that were statistically significant in the binary logistic regression analyses were further examined using multivariable logistic regression. Thus, twelve variables (alcohol drinking, sex of a child, history of stillborn, birth order, vitamin conception, history of pesticide exposure, diabetic mothers, lack of folic acid use, history of injury and BD, caffeine use, and lack of ANC) whose *p*-value less than 0.25 in the crude odds ratio were entered into the multivariable logistic regression model to identify the predictor variables associated with externally visible BDs.

As a result, alcohol consumption, lack of ANC, history of stillbirth, pesticide exposure, family history of BDs, lack of folic acid conception during pregnancy, and child’s gender were statistically significant at *p*-values less than 0.05. However, in this study, being a diabetic mother, caffeine and drug use during early pregnancy, vitamin conception, and birth order were not statistically significant (Table 3). According to Table 3, women who drank alcohol during pregnancy were 6.575 times more likely to have a child with BD (AOR = 6.575; 95% CI: 3.102, 13.937) than their counterparts. Women who had an ANC follow-up during pregnancy were 2.794 times more likely to have a child with BD than those who had an ANC follow-up (AOR = 2.794; 95% CI: 1.333, 5.859). Mothers of the stillbirth child were 3.967 times more likely to have a child with a BD than the mothers of live birth (AOR = 3.967; 95% CI: 1.772, 8.881). Women who were exposed to pesticides during early pregnancy were 4.84 times more likely to have a child with BD than those who were not exposed to pesticides (AOR = 4.840; 95% CI: 1.375, 17.034). Women who had a previous history of BD were 4.853 times more likely to have a child with BD than had no previous history of BD (AOR = 4.853; 95% CI: 1.492, 15.788). Furthermore, women who did not take folic acid supplementation during early pregnancy were 4.324 times more likely to have a child with BD than those who did (AOR = 4.324; 95% CI: 2.062, 9.067) (Table 3).

**Table 3:** Bivariate and multivariable logistic regression analysis of risk factors associated with externally visible birth defects at ACSH, Adama, Ethiopia, 2020

Variable		Case(*n* = 63)	Control(*n* = 252)	COR (95% CI)	AOR (95% CI)
		N (%)	N (%)		
Alcohol Use	Yes	33(52.4%)	49(19.4%)	4.55(2.540, 8.177)	6.57(3.102,13.937) *
	No	30(47.6%)	203(20.6%)	1	1
Diabetic mother	Yes	9(14.3%)	15(6%)	2.633(1.095,6.334)	2.197(0.636,7.585)
	No	54(85.7%)	237(94%)	1	1
Lack of ANC	Yes	28(43.4%)	67(26.6%)	2.209(1.249,3.907)	2.794(1.333,5.859) *
	No	35(55.6%)	185(73.4%)	1	1
History of stillborn	Yes	24(38.1%)	33(13.1%)	4.084(2.183,7.641)	3.967(1.772,8.881*
	No	39(61.9%)	219(86.9%)	1	1
Caffeine Use	Yes	9(14.3%)	17(6.8%)	2.304(0.975,5.447)	2.370(0.810,6.936)
	No	54(85.7%)	235(93.2%)	1	1
Drug use	Yes	4(6.3%)	8(3.2%)	2.068(0.602,7.099)	2.276(0.486,10.649)
	No	59(94.7%)	244(96.8%)	1	1
Vitamins consumption	Yes	13(20.6%)	32(12.7%)	1	1
	No	50(79.4%)	220(87.3%)	0.559(0.274,1.142)	0.562(0.226,1.395)
Exposure to pesticide	Yes	9(14.3%)	11(4.7%)	3.652(1.442,9.246)	4.84(1.375,17.034) *
	No	54(85.7%)	241(95.3%)	1	1
Family history CAs	Yes	10(15.9%)	18(7.1%)	2.453(1.071,5.617)	4.85(1.492,15.788) *
	No	53(84.1%)	234(92.9%)	1	1
Lack of folic acid	Yes	47(24.6%)	105(41.7%)	4.112(2.212,7.646)	4.324(2.062,9.067) *
	No	16(25.4%)	147(58.3%)	1	1
Birth order	1–4	53(84.1%)	182(72.2%)	1	1
	> 4	10(15.9%)	70(27.8%)	0.491(0.236,1.018)	0.493(0.200,1.213)
Sex of children	Male	42(66.7%)	122(48.4%)	2.131(1.194,3.803)	2.067(1.009,4.238) *
	Female	21(33.3%)	130(51.6%)	1	1

*Note* * statistically significant at *p*-value < 0.05; 1, Reference *Abbreviation* ANC, Antenatal Care; AOR, Adjusted Odds Ratio; CAs, Congenital Anomalies; CI, Confidence Interval; N, Frequency; n, Sample Size

## Discussion

In this study, the most common cases of externally visible BDs were the central nervous system. This finding is consistent with studies done in South India, South Africa, Morocco, Tanzania, Northwest Ethiopia, and Southeast Ethiopia [3, 6, 13–16]. However, this finding is inconsistent with the studies conducted in the United States, England, Ogbomosho, and Nigeria that reported the cardiovascular system was the most commonly affected system, followed by the digestive system [17–20]. This finding is also different from studies done in Egypt and India, which reported the most common CAs were GIT and musculoskeletal system [7, 14]. The discrepancy might be due to sampling methods. In this study, retrospective study methods were used to identify the determinants of externally visible BDs using secondary data. However, those studies which were different from the current study used a prospective study method to identify associated risk factors and they include both internally and externally visible BDs.

This study indicated that alcohol consumption during pregnancy was found to have a strong association with the occurrence of BDs. This finding is in line with various studies conducted in Southeast Ethiopia (Arsi), Southwestern Ethiopia, and the Amhara region [3, 6, 12, 21]. Another study also showed that pregnant mothers drinking any amount of alcohol during early pregnancy had direct effects on the growth and morphogenesis of fetuses [3].

According to the present study, mothers who lack ANC were 2.79 times more likely to have a child with BD than mothers who had ANC. This finding is in line with the report from Tanzania, and Iraq that reported CAs were significantly associated with inadequate ANC [9, 15]. This association is also supported by another study in Pakistan where only 32.3% of mothers of babies with CAs had ANC irrespective of the stage of pregnancy [22].

In this finding, more stillbirths (71.9%) were observed in cases as compared to the controls (11.66%). It is similar to the study conducted in the United States, which reported the overall CA specific stillbirth risk was found to increase among affected fetuses over the occurrence of stillbirth in the general population [21]. This finding also agrees with a study done in Iran where malformations were seen more significantly in stillbirths as compared to live birth and a study from India where congenital malformation was significantly high in stillborn babies as compared to live-born babies [23, 24].

According to this study, drug use during early pregnancy had no significant associations with BDs. This finding is different from a study conducted in Northern Ethiopia where Infants born from mothers who took drugs during pregnancy were 3.55 more likely to have a BD compared to infants born to mothers who did not take any type of the drug during pregnancy [3]. This difference might be due to the sociodemographic characteristics of the mothers of a child. In this study, the majority of mothers were living in urban areas and employed whereas the report from northern Ethiopia showed that most of the participants were from rural areas and farmers. People who live in urban areas are more informed about the usage of the drug than those who live in rural areas; because in rural areas most people use unprescribed drugs without an order from a physician and traditional medicine.

In this study, mothers who had been exposed to pesticides during pregnancy were 4.84 times more prone to have infants with BDs compared to their corresponding counterparts. This result is supported by studies done in the Amhara region, Southeastern Ethiopia, and Southwestern Ethiopia, which found that maternal pesticide exposure during early pregnancy was strongly linked to the occurrence of CAs [6, 12, 21]. This finding is also consistent with a study from the Bale zone of Ethiopia, which reported pregnant women who had been exposed to pesticides were twice as likely as their counterparts to have congenital malformations [11].

This study indicated that infants born from mothers who had a previous history of BDs were 4.8 times more likely to have BDs compared to infants born from mothers who did not have a previous history of a child with BDs. This finding is supported by studies done in Pakistan and the United Kingdom where families with a familial condition with pregnancy to CAs are concerned about the risk of recurrence in future pregnancies [22, 25].

In this study, there was no strong link between smoking and the occurrence of BDs. This finding is similar to the studies conducted in Tanzania, Nigeria, Iraq, and Ethiopia [9, 12, 15, 17]. Unlike this finding, studies conducted in Ethiopia revealed a strong association between smoking and the risk of having a child with CAs [12, 21]. This difference might be due to socioeconomic, religious, and cultural differences. For instance, in Iraq the number of smokers has increased over the last three decades due to psychological and associated factors with post-war conflicts [26]. In Egypt, it was estimated that twenty billion people smoke tobacco annually [27].

There was a strong association between maternal folic acid supplementation during early pregnancy and the occurrence of BDs in this study. Mothers of children who received folic acid supplementation during early pregnancy were significantly less likely to have a child with BDs. Mothers who did not receive folic acid supplementation during early pregnancy had a fourfold increased risk of having a child with BDs. This finding is consistent with studies conducted in Nigeria, Iraq, and Ethiopia, where folic acid supplementation was deficient [1, 17, 28]. This result was also in accordance with some studies done in Sudan and Ethiopia that reported maternal folic acid supplementation significantly decreased the prevalence of NTDs [1, 29]. Recent evidence has shown that daily supplements of 400 micrograms of folic acid started 3 months before conception prevent up to 70% of these defects [30]. Even though the WHO recommends periconceptional folic acid supplementation, studies showed that many women still do not follow the recommendations, particularly women of low socioeconomic status [31]. Similarly, the Ministry of Health of Ethiopia promotes folic acid supplementation to all pregnant women, but folic acid usage is still low. This is also true in our study; though 17% of the participants had preconception care, only 7.8% were supplemented. This could be due to a lack of healthcare facilities in this area of study and Ethiopia, as well as community resistance due to a lack of knowledge about the importance of folic acid supplementation.

Consanguinity had no strong association with the occurrence of BDs in this study. This finding is in contrast to one study that reported consanguinity was a major risk factor for the development, where 89% of spinal Bifida was a relative parent compared with 67% of controls [29]. Close consanguinity is a known risk factor for CAs, as well as Mendelian conditions such as inborn errors of metabolism (occurring 1 in 770 births in this study), as confirmed in prior reports from Saudi Arabia and the world literature [32]. The discrepancy might be due to religious and cultural differences. This is because consanguineous marriage is widely practiced in Saudi Arabia where the majority of people in Saudi Arabia were Muslim and consanguineous marriage were culturally allowed while Ethiopia is a multicultural and religious country where consanguineous marriage is not allowed.

This study revealed that males were 2.131 times more prone to have BDs than females. This finding agrees with studies done in Iran, Brazil, and Ethiopia [1, 4, 5, 8, 11]. This could be due to chromosomal abnormalities and gene mutations, which are common in both males and females under similar circumstances [11]. However, more research is required to justify the condition.

Most of the participants in this study, who had a child with BDs, were less than 35 years old, which was different from studies in Pakistan where higher maternal age was a well-known risk factor [22]. This study was also different from a study in Iraq which reported CAs showed low incidence with ages less than 20 years and high with ages between 20 and 35 years old [28]. This could be explained by the fact that the study was conducted in a hospital setting where only those mothers who need special care were referred to this hospital and younger women gave birth at health institutions than elders. Still, deliveries are conducted at home traditionally in this study setting and most of them are elders who are illiterate and ashamed to give birth at health institutions. Youngers are more educated and informed about health services and risk factors related to pregnancy.

### Limitations of the study

This study was a retrospective study that covered one medical center. A long-term follow-up and prospective study are required to assess all abnormalities, including functional abnormalities, which were not the case in the present study. We were unable to review all case files of maternal admission and had adequate information on the maternal characteristics and risk factors. This study could not consider the internal congenital anomaly like congenital heart disease due to lack of health facilities so it was difficult to compare with the published researches of birth defects. This was also a hospital-based study, so its findings may not apply to the general population.

## Conclusion

In this study, BDs appear to be the most important causes of perinatal deaths and hence it becomes necessary to keep on account its associated risk factors. Of the associated factors with perinatal death with externally visible BDs; alcohol conception, exposure to the pesticide, previous history of stillbirth, and history of BDs were found associated with the externally visible BDs. ANC and folic acid supplementation during pregnancy were identified to have a protective effect against the occurrence of perinatal death with BDs. Public health experts and communities must pay close attention to the risk factors for BDs and their preventive strategies.

## Data Availability

All data obtained are available in this manuscript. However, any reasonably required data will be available per the corresponding author on request.

## Abbreviations

AOR: Adjusted Odds Ratio
BD: Birth Defect
CA: Congenital Anomaly
COR: Crude Odds Ratio
ICD: International classification of disease
MCEE: Maternal and child epidemiology estimation group
NTD: Neural tube defect
ODK: Open data kit
WHO: World Health Organization
